# DNA methylation of noncoding RNAs: new insights into osteogenesis and common bone diseases

**DOI:** 10.1186/s13287-020-01625-7

**Published:** 2020-03-06

**Authors:** Liyuan Yu, Kai Xia, Xiao Cen, Xinqi Huang, Wentian Sun, Zhihe Zhao, Jun Liu

**Affiliations:** 1 State Key Laboratory of Oral Diseases & National Clinical Research Center for Oral Diseases, West China Hospital of Stomatology, Sichuan University, Chengdu, Sichuan China; 2 Department of Orthodontics, West China Hospital of Stomatology, Sichuan University, No. 14, 3rd Section, South Renmin Road, Chengdu, 610041 Sichuan China; 3 Department of Temporomandibular Joint, West China Hospital of Stomatology, Sichuan University, Chengdu, Sichuan China

**Keywords:** DNA methylation, Noncoding RNAs, Osteogenesis, Bone

## Abstract

Bone diseases such as osteoarthritis, osteoporosis, and bone tumor present a severe public health problem. Osteogenic differentiation is a complex process associated with the differentiation of different cells, which could regulate transcription factors, cytokines, many signaling pathways, noncoding RNAs (ncRNAs), and epigenetic modulation. DNA methylation is a kind of stable epigenetic alterations in CpG islands without DNA sequence changes and is involved in cancer and other diseases, including bone development and homeostasis. ncRNAs can perform their crucial biological functions at the RNA level, and many findings have demonstrated essential functions of ncRNAs in osteogenic differentiation. In this review, we highlight current researches in DNA methylation of two relevant ncRNAs, including microRNAs and long noncoding RNAs, in the initiation and progression of osteogenesis and bone diseases.

## Introduction

Bone is the primary connective tissue of the human body and undergoes constant renewal and remodeling during growth, damage, and normal homeostasis. Nowadays, bone diseases, including osteoarthritis, osteoporosis, and bone tumor, become prevalent and severe public health threats. However, bone regeneration ability declines with age and changes in some pathologic conditions, eventually leading to the reduction of bone density or osteoporosis. Osteogenic differentiation is a complex process involving tight coordination of proliferation and differentiation of different cells, synthesis, and mineralization of extracellular matrix [1]. It is achieved through a multi-tiered regulatory system by transcription factors, cytokines, many signaling pathways, and epigenetic modulation [2].

Epigenetic modifications, such as methylation and histone modifications, implicate in the heritable genetic changes without DNA sequence alteration often related to human disease [3]. DNA methylation is a kind of stable epigenetic modifications and refers to the addition of a methyl group (CH3) to the C-5 position of cytosine, which usually occurs in CpG islands [4]. CpG islands enrich cytosine and guanine sequences and account for 1% of the genome [5, 6]. Approximately 60% of CpG islands are found in gene promoters and are usually demethylated in normal cells [5, 7, 8]. DNA methyltransferase (DNMT) enzymes, including DNMT1, DNMT3A, and DNMT3B, can catalyze DNA methylation, changing the DNA epigenetic status [9]. DNMT1 is highly related to maintaining DNA methylation, while DNMT3A and DNMT3B have the ability to promote the DNA methylation rate [10]. The aberrant DNA methylation statuses play an essential role in the pathological process of some diseases [11]. Previous studies determined that DNA methylation was involved in the process of osteoblastic differentiation [12] and osteoclast formation [13], as well as the transformation of osteoprogenitor cells into osteoblasts [14].

Noncoding RNAs (ncRNAs) are RNA molecules transcribed from the genome without open reading frame and protein-coding ability, but they can perform their crucial biological functions at the RNA level [15]. According to the transcript size, ncRNAs can be roughly divided into small noncoding RNAs and long noncoding RNAs (lncRNAs). Small noncoding RNAs are < 105 nucleotides in length, including microRNAs (miRNA), transfer RNAs (tRNAs), and circular RNAs (circRNAs), while lncRNAs were longer than 200 nucleotides [16]. Recently, ncRNAs were implicated in several genetic, biological, and cellular processes, including cell cycle control, epigenetic modification, cell differentiation, and stem cell regulation [17, 18]. In this review, we highlight the current studies of two significant ncRNAs, including lncRNAs and miRNAs, in pathogenesis and progression of osteogenesis and bone disease.

## Cells differentiation

Under the stimulus of different microenvironments, mesenchymal stem cells (MSCs) have the potential to differentiate into osteoblasts, adipocytes, or chondrocytes [19], which are strictly regulated by cellular signaling molecules, cytokines, transcriptional factors, and multiple genes [20]. During embryonic development and bone regeneration in fracture healing, bone formation occurs through two interrelated mechanisms: intramembranous osteogenesis and endochondral osteogenesis [21]. In the process of intramembranous osteogenesis, MSCs directly differentiate into osteoblasts. Osteoblast differentiation is the primary step in the process of bone formation, and its regulatory pathways include a variety of signaling pathways such as bone morphogenetic protein (BMP), runt-related transcription factor 2 (RUNX2), transforming growth factor-beta (TGF-beta), and mitogen-activated protein kinase (MAPK) signaling pathway, as well as various transcription factors regulated by ncRNAs [22]. However, during endochondral osteogenesis, MSCs first differentiate into chondrocytes and form chondroid tissue, which is eventually replaced by bone tissue [23].

Many studies have put forward that the differentiation of MSCs into osteoblasts and adipocytes is the opposite. A variety of genes have been proposed to participate in the cell fate decision. For example, in the regulation of some genes such as PPARG and CXCL12, MSCs differentiate into adipocytes and promote adipogenesis, while inhibited osteogenesis [24, 25]. Besides, the researchers found that Wnt/β-catenin was activated in the differentiation of MSCs towards osteoblasts whereas inhibited in the differentiation towards adipocytes [26]. As with the idea that “bone loss is fat gain” [27], osteogenic and adipocytic differentiations are a two-way balance process that, if broken, can result in some human diseases, such as osteoporosis [28, 29].

A deeper understanding of the regulatory mechanism underlying cell lineage of MSCs is helpful to explore the occurrence and development of osteogenic diseases. In recent years, considerable numbers of studies have reported the function of DNA methylation in the MSCs differentiation. For example, Sorensen et al. [30] found hypermethylation of lineage-specific promoters was associated with the differentiation restriction of MSCs. Likewise, a study by Malvicini et al. [31] revealed that the downregulated OCT4 in MSCs triggered hypermethylated modifications, further impairing the ability of MSCs to differentiate into osteoblasts and adipocytes. A new regulatory mechanism has been identified that ncRNAs, such as lncRNAs and miRNAs, can be potential triggers in the decision of cell fates by methylated modification. For example, lncRNA Plnc1 was reported to mediate the differentiation of bone marrow stromal cells (BMSCs) into adipocytes by DNA methylation [32], and miRNA-455-3p changed the methylation status of chondrogenic-specific genes during the differentiation of human bone marrow mesenchymal stem cells (hBMSCs) towards chondrocytes [33], which would be discussed in more detail in later sections.

## Osteogenic differentiation

### MicroRNAs

MicroRNAs (miRNAs) are single-stranded ncRNAs with approximately 22 nucleotides, and multiple miRNAs have been found to regulate the expression of osteogenic-related genes at the post-transcriptional level. DNA methylation of miRNAs was determined to regulate the development of many human diseases. For example, miR-34b was reported to affect leukemia cell proliferation by DNA methylation [34]. The latest research also found that miRNAs had this particular regulation function in osteogenic differentiation (Table 1).

**Table 1:** DNA methylation of ncRNAs in the osteogenic differentiation

ncRNAs	Gene ID	DNA methylation and effects	References
miRNA	miR-149	Hypermethylation of miR-149 regulated the osteogenic differentiation of MSCs via SDF-1/CXCR4 signaling.	Li et al. [35]
miRNA	miR-29b	MiR-29b targeted DNMT1 and led to the methylation modification in the Notch1 promoter, which affected the Notch signaling pathway and regulated the osteogenic differentiation in BMSCs.	Liu et al. [36]
miRNA	miR-342-3p	Hypomethylation in the promoter of EVL promoted the miR-342-3p expression in osteogenic differentiation of hMSCs and human pre-osteoblast.	Han et al. [37]
lncRNA	H19	H19 has a strong osteogenic effect via the NOTCH1 pathway, and hypomethylation in the H19 promoter was associated with the high expression of H19.	Hadji et al. [38]
lncRNA	H19	Hypermethylation in the promoter of H19 by DNMT1 resulted in the low expression of H19 and suppression of the ERK signaling in disuse osteoporosis.	Li et al. [39]
lncRNA	ANRIL	DNA methylation of CDKN2A promoter mediated ANRIL expression and altered the binding of the transcription factor, which was inversely associated with bone size, bone density, and mineralization.	Curtis et al. [40]

*ncRNAs* noncoding RNAs, *DNMT* DNA methyltransferase enzymes, *BMSCs* bone marrow stromal cells, *hMSCs* human mesenchymal stem cells

**Fig. 1:**
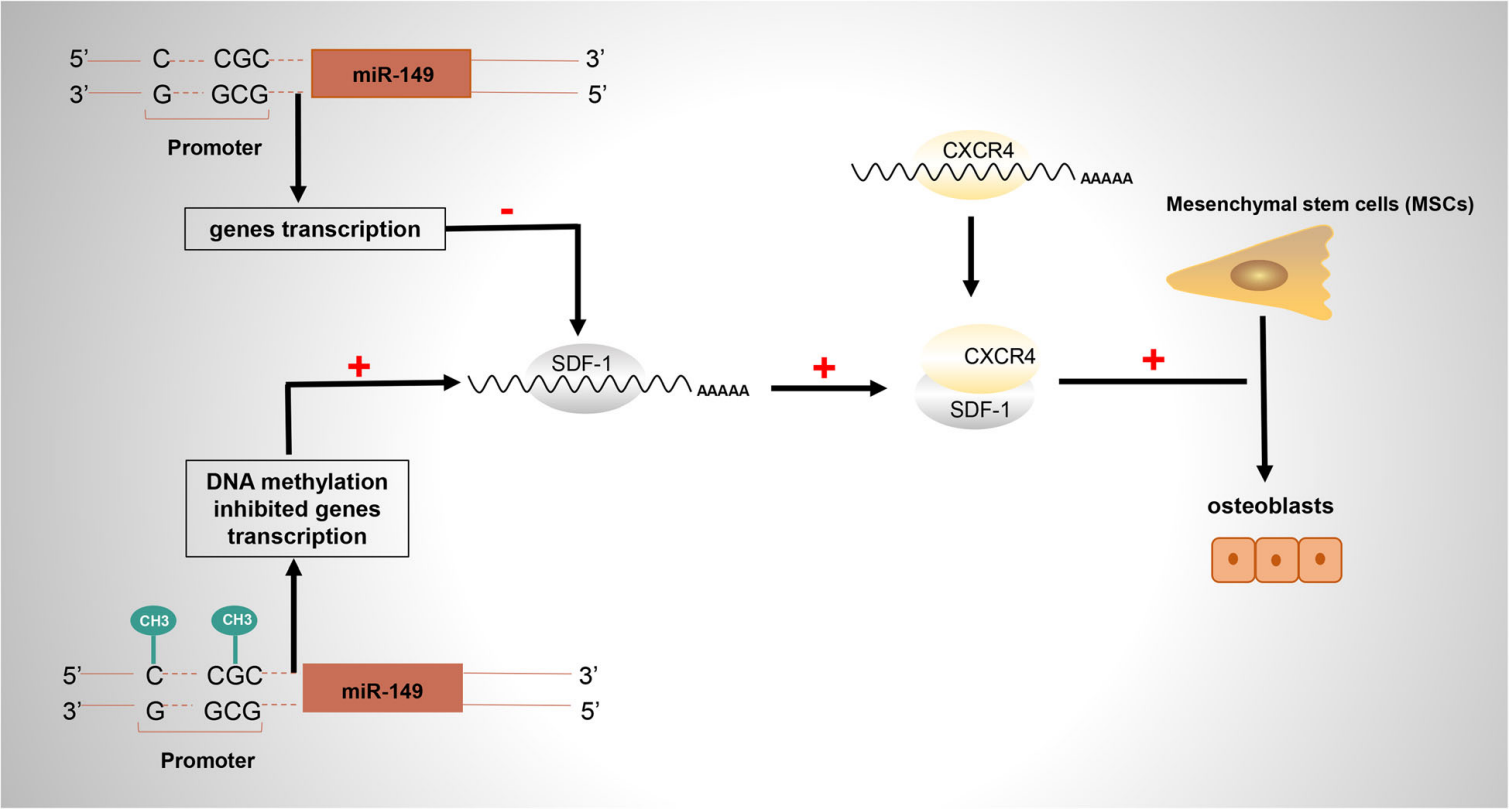
Hypermethylation at the CpG sites of miR-149 promoter inhibited the expression of miR-149, thereby eliminating the inhibitory effect on SDF-1 and activating the SDF-1/CXCR4 signaling, which ultimately induced osteogenic differentiation of MSCs [35]

In 2019, Li et al. reported high-frequency methylation of miR-149 regulated the osteogenic differentiation of MSCs. They found miR-149 directly targeted SDF-1 and regulated SDF-1/CXCR4 signaling during MSCs osteogenic differentiation. When treated with the methyltransferase inhibitor 5′-AZA-2′-deoxycytidine (5′-AZA), the methylation levels were declined and the expression of miR-149 were elevated. It revealed that miR-149 regulated MSCs osteogenic differentiation through epigenetic modifications (Fig. 1) [35].

Another research confirmed that miR-29b targeted DNMT1 and led to the methylation modification in the Notch1 promoter, which affected the Notch signaling pathway and regulated osteogenic differentiation in BMSCs of systemic lupus erythematosus mice. When miR-29b was overexpressed, DNMT1 mRNA expression was downregulated, resulting in demethylation in the promoter of Notch1. Hypomethylated modification promoted Notch1 expression, and the activation of Notch signaling decreased osteogenic differentiation of BMSCs [36].

Enah/Vasp-like (EVL) is an actin-related protein of the Ena/VASP family, which involved in various processes related to cell polarity and cytoskeletal remodeling, including axon guidance and cell migration [41]. It was reported that EVL contained a CpG island in the promoter region, and the CpG island aberrant methylation inhibited miR-342 expression in human colorectal cancer, which was a transcription product of EVL intron [42]. Another article confirmed the effect of miR-342-3p in osteogenic differentiation and explored the regulation mechanism between miR-342-3p and DNA methylation of its hosting gene EVL [37]. They found hypomethylation in the promoter of EVL promoted the miR-342-3p expression in osteogenic differentiation, while hypermethylation associated with low expression of miR-342-3p in the undifferentiated group.

### lncRNAs

lncRNA is a kind of ncRNAs longer than 200 nucleotides and possesses mRNA-like characteristics, including 5′ cap and 3′ polyA tail, but no protein-coding ability [43]. Emerging evidence shows that lncRNAs are aberrantly methylated in osteogenic differentiation (Table 1) and thus contributed to the pathogenesis and progression of bone disease.

lncRNA H19 is transcribed from maternally inherited genes and is a crucial regulator of osteogenic differentiation, which has an underlying association with bone-related diseases [44]. As an imprinted gene, DNA methylation changes of H19 can also lead to differential expression of H19 and involves in bone diseases. In 2016, Hadji et al. found a reduction of DNA methylation in lncRNA H19 promoter, and its expression level was increased in calcific aortic valve disease [38]. The researchers subsequently showed a strong osteogenic effect of H19 via negatively regulating the NOTCH1 pathway. In 2018, Liu et al. confirmed that DNMT1 expression was significantly enhanced in disuse osteoporosis and resulted in 5-methylcytosine cumulation in the H19 promoter, which accompanied by low expression of lncRNA H19 and suppression of the ERK signaling [39]. This evidence revealed the crucial function of H19 methylation in controlling skeletal metabolism.

lncRNA ANRIL was reported to be involved in regulating bone development as well. CDKN2A promoter was identified to contain CpG regions and demonstrated that DNA methylation changes in these sites mediated lncRNA ANRIL expression and altered the binding of the transcription factor, which was inversely associated with bone size, bone density, and mineralization [40].

## Adipogenic differentiation

In recent years, the role of DNA methylation of miRNA in adipocyte differentiation has been investigated. For example, in 2018, Miranda et al. detected miRNA expression profile in the obese mice, and miRNA-30 family (miRs 30a-5p, 30c-5p, and 30e-5p) was identified to be downregulated. Further research revealed that low expression of miRNA-30 eliminated Notch1-mediated inhibition of adipogenic differentiation. More importantly, they found a high level of DNA methylation in the CpG island of miRNA-30, indicating that DNA methylation alteration of miRNA-30 might be associated with obesity [45].

As previously described, lncRNA Plnc1 had an active effect on adipocyte formation by DNA methylation. The knockdown of Plnc1 inhibited BMSCs differentiating towards mature adipocytes. However, overexpression of Plnc1 was observed in adipose tissue and induced adipogenic differentiation via PPAR-λ2. The biological mechanism indicated that Plnc1 increased the transcriptional activity of PPAR-λ2 through mediating the CpG region hypomethylation in the process of adipogenesis [32].

## Chondrogenic differentiation

A previous study reported that the potential role of DNA methylation in miRNA regulated chondrogenic differentiation under hypoxic conditions. MiR-124 was downregulated and promoted the expression of Sox9 by targeting NFATc1 during chondrogenesis in hypoxia [46]. They found CpG islands in the miR-124 promoter and detected hypermethylation level of the promoter under hypoxic conditions, which was significantly decreased by treating with 5′-AZA. Further experiments suggested that low methylation levels with 5′-AZA elevated the miR-124 expression and impeded the initiation of chondrogenic differentiation.

It was reported that miR-455-3p directly targeted the 3′-UTR of DNMT3A and regulated the process of chondrogenic differentiation in hBMSCs by altering the methylation levels of genes associated with cartilage development. Most of these genes, including FOXO3A, SMAD3, COL11A1, and SOX6, were hypomethylated and involved in the P13K-Akt signaling pathway [33], revealing the hypomethylated signaling pathway was a crucial regulator of chondrogenic differentiation.

The P13K-Akt signaling pathway can control many differentiation processes including chondrocyte proliferation, osteogenic differentiation, adipogenic differentiation, and cell apoptosis [47–49]. Various studies have proposed the interacted regulation role between PTEN and P13K-Akt signaling in many diseases [50, 51]. In 2019, Shen et al. found different DNA methylation levels in the promoter of PTEN in BMSCs and dental pulp mesenchymal stem cells (DP-MSCs), which related to the lineage commitment [52]. Hypomethylation of PTEN was mediated by DNMT3B and downregulated PTEN expression, which promoted P13K-AKT signaling and induced BMSC adipogenic differentiation. However, high expression of PTEN was observed, and the P13K-Akt pathway was downregulated in DP-MSCs, which promoted the osteogenic differentiation of DP-MSCs. A large number of miRNAs have been reported to regulated osteogenesis by the PTEN/P13K-Akt pathway. For example, miRNA-21 and miRNA-181a/b-1 were determined to promote osteogenic differentiation by modulating the PTEN/PI3K/AKT signaling pathway [53, 54]. It is speculated that these miRNAs are likely to regulate osteogenic differentiation by altering DNA methylation of the PTEN/PI3K/AKT signaling pathway. Although further studies are required to verify the speculation, it sheds light on the progress for novel therapeutic strategies in the prevention or treatment of bone diseases.

## DNA methylation of ncRNAs in common bone diseases

### Osteoarthritis

Osteoarthritis (OA) is a prevalent joint chronic disease, which is characterized by cartilage degeneration, including fibrosis of synovial membrane, synovial inflammation, and subchondral bone reconstruction, resulting in structural changes and function loss in articular cartilage of the hip, knee, and hand [55]. GWAS research revealed epigenetics altered OA-related gene expression without changing the DNA sequence, including DNA methylation, histone acetylation, and ncRNAs [56].

It was the first time to screen methylated genes in health and OA synovial cells and framed regulatory networks based on miRNAs and related methylated genes, opening the research of ncRNAs and methylation in OA [57]. Zhang et al. performed genome-wide DNA methylation to study the development of knee osteoarthritis; identified DNA methylation changes of EMX2OS, the chain encoding lncRNAs; and predicted many miRNAs regulating methylated genes, such as miR-130a-3p and miR128, revealing DNA methylation of ncRNAs may in part mediate OA [58].

In 2019, Papathanasiou et al. reported the high-frequency methylation of miR-140 and miR-146a in the CpG island inhibited the miR-140-5p and miR-146a expression and reduced the binding with SMAD-3 and NF-kB in both osteoarthritic chondrocytes and synoviocytes, which was reversed by treating the 5′-AZA [59]. Another study confirmed MMP-13 was inhibited by 5′-AZA, and TET-1 was downregulated in OA chondrocytes, which was reported to induce DNA methylation in many biological processes. Besides, miR-370 or miR-373 could target SHMT-2 (serine hydroxymethyltransferase) or ECP-2 (methyl-CpG-binding protein) and regulated MMP-13 expression in OA chondrocytes, suggesting that these miRNAs might mediate dysregulation of methylation in OA events [60].

In 2019, lncRNA XIST was reported to mediate the degradation of collagen in OA by inducing hypermethylation of TIMP-3 and downregulating the expression of TIMP-3 [61]. Kim et al. determined that miR-101 was downregulated, which could target DNMT-3B and changed the methylation status of integrin-α1 in OA. Additionally, lncRNA HOTTIP was overexpressed in OA chondrocyte, and HoxA13 was inhibited, thereby suppressing integrin-α1. The results suggested that miR-101 and lncRNA HOTTIP contribute to OA progression by epigenetic modification of integrin-α1 [62].

In addition, DNA-methylated modification may provide a potential therapeutic strategy for OA. It is generally recognized that a low methylation level in the promoter of IL-1β is associated with the initiation and progression of OA [63]. Besides, it was confirmed that glucosamine (GlcN) can reverse hypomethylation in the CpG island of IL-1β promoter and inhibit the expression of IL-1β in OA, revealing that DNA methylation alteration could intervene in the process of OA and could be a prospective therapeutic approach [64]. Several ncRNAs have been determined to regulate IL-1β in OA, suggesting that ncRNAs may participate in DNA methylation of IL-1β in the pathogenesis and progression of OA. For example, miR-204 promoted cartilage degradation in OA via targeting IL-1β [65], and lncRNA HOTAIR could regulate IL-1β function in the pathogenesis of OA [66]. Further studies are required to elucidate the underlying regulatory mechanisms.

### Osteosarcoma

Emerging evidence has revealed the interaction between lncRNA and miRNA and demonstrated the dysregulation of ncRNAs implicated in the pathogenesis and progression of cancer, including osteosarcoma (OS) [67]. OS is the most frequent primary tumors of the bone, which are derived from mesenchymal cells and produce immature bone and osteoid [68]. Recent researches offered new insights into DNA methylation in osteosarcoma pathogenesis, progression, and therapy.

The relationship between aberrant methylation and miRNAs has been involved in OS. For example, miR-485-3p was demonstrated to directly interact with the 3′-UTR of CtBP1, while the expression of miR-485-3p was associated with the DNA methylation of CpG islands in its promoter. When treating with 5′-AZA, miR-485-3p was upregulated to inhibit OS cell development by reducing CtBP1 expression [69]. High methylation levels in the CpG sites of miR-7 promoter were decreased by treating 5′-AZA, thus promoting miR-7 expression in OS cells. The overexpression of miR-7 inhibited OS oncogenic phenotypes via targeting IGF1R [70]. MiR-370 was significantly downregulated by DNA methylation in OS cells, which eliminated the inhibition of FOXM1 and β-catenin and promoted the Wnt/β-catenin pathway [71]. Besides, miR-142 was downregulated in OS cells with hypermethylated modification in the CpG island, indicating the association between aberrant methylation and OS [72].

It has been reported that the occurrence of osteosarcoma is also related to the methylation alteration of lncRNAs. Li et al. identified that lncRNA HOTAIR was upregulated in OS cells and significantly inhibited CDKN2A expression by hypermethylated modification of the CDKN2A promoter. They found the downregulation of HOTAIR suppressed the DNMT1 expression and thereby led to the changes in DNA methylation [73]. Their further research indicated that HOTAIR regulated the DNMT1 expression via inhibiting miR126 expression. This article enriched a new insight into the regulation mechanism and interaction between ncRNAs and DNA methylation in OS and wound and provided a novel strategy in treating OS patients.

DNA modification is expected to be a treatment for OS. A study found that demethylation promoted miR-129-5p overexpression and inhibited the metastasis and invasion of OS via targeting valine-containing protein (VCP) [74], which could be a treatment for OS in the future. In order to study the recurrence and survival of patients with OS, a recent study described the profile of miRNA expression and found that most of these prognostic miRNAs were sited in 14q32, which affects the gene expression through DNA methylation [75]. It is reported that the human 14q32 location encoded more than 40 miRNAs, including imprinted genes that were important in osteogenic differentiation and inhibiting cancer [76]. The degree of DNA methylation in the differentially methylated regions located in the 14q32, which regulated the imprinted genes, participated in the development of OS and could predict the prognosis of OS, suggesting that the strategy of repairing DNA modification for treating OS might be possible [77].

## Conclusions

DNA methylation can affect gene expression and change gene function by altering the methylation status and is associated with various human diseases. Plenty of evidence have revealed that DNA methylation of different genes plays a significant role in bone development, homeostasis, and osteocyte activity. Recently, there have been considerable researches into the role of ncRNAs in cancer and bone diseases as a result of epigenetic alterations. In this review, we present several studies on the effects of DNA methylation on CpG island of ncRNAs promoters, which consequently influenced the osteogenic function of ncRNAs. Some ncRNAs can regulate the expression of DNA methyltransferase enzymes and other DNA methylation-related enzymes and then change the methylation level of osteogenic related genes. The complicated mechanisms of DNA methylation and ncRNAs in osteogenesis are essential to understand the pathological process of bone-related diseases and remain unclear.

Recently, DNA methylation has been used as a therapeutic method for cancer. DNMT inhibitors (DNMTi) are generally classified as nucleoside and non-nucleoside [78]. Azacitidine is a kind of nucleoside inhibitor, and low-dose azacitidine has shown clinical benefit in the treatment of myelodysplastic syndrome (MDS) in the methylation mechanism, and FDA has approved azacitidine drugs for the treatment of MDS [79]. Meanwhile, zebularine, another nucleoside inhibitor, has also been confirmed to be effective in treating bone marrow disease [80]. DNMTi drugs may also have a therapeutic effect on bone diseases. Although the relevant research is minimal, DNA methylation of ncRNAs is expected to be a promising therapeutic strategy for bone diseases.

## Data Availability

Not applicable.

## Abbreviations

5′-AZA: 5′-AZA-2′-deoxycytidine
BMP: Bone morphogenetic protein
BMSCs: Bone marrow stromal cells
CH3: Methyl group
circRNAs: Circular RNAs
DNMT: DNA methyltransferase enzymes
DNMTi: DNMT inhibitors
DP-MSCs: Dental pulp mesenchymal stem cells
EVL: Enah/Vasp-like
GlcN: Glucosamine
hBMSCs: Human bone marrow mesenchymal stem cells
lncRNAs: Long ncRNAs
MAPK: Mitogen-activated protein kinase
MDS: Myelodysplastic syndrome
MECP-2: Methyl-CpG-binding protein
miRNA: MicroRNAs
MSCs: Mesenchymal stem cells
ncRNAs: Noncoding RNAs
OA: Osteoarthritis
OS: Osteosarcomas
RUNX2: Runt-related transcription factor 2
SHMT: Serine hydroxymethyltransferase
TGF-beta: Transforming growth factor beta
tRNAs: Transfer RNAs
VCP: Valine-containing protein
